# Parliament and the Profession

**Published:** 1917-03-10

**Authors:** 


### PARLIAMENT AND THE PROFESSION.

### Industrial Employment—Effect on Women's Health.

Mr. Rowntree asked the Prime Minister if he would consider the advisability of setting up a special expert commission, as a preliminary to the work of demobilisation, to inquire into the effect of different employments on the health and physique of women and girls. The Home Secretary stated that the effects of the new industries introduced during the war are being carefully noted by the inspectors of the Factory Department, and the information collected will be at the disposal of the Reconstruction Committee which is considering the employment of women after the war. Sir George Cave did not think any further expert inquiry is needed at the present time.

### Officers' Convalescent Home.

Mr. Macpherson informed Mr. MacVeagh that officers at the convalescent home at the Great Central Hotel are prohibited from leaving the hospital before 1 p.m. and from going to matinées without permission. These rules are applicable to all military hospitals.

### Epileptic Soldiers.

Sir A. Griffith-Boscawen, answering on behalf of the Minister of Pensions, informed Mr. King that no precise information is available as to the number of persons who have become epileptic subsequent to enlistment, but that he is advised that it is not large. Treatment and training are being carefully considered. A scheme has been approved for building additions to the accommodation at the Chalfont Colony, and a special committee on institutional treatment, who have secured the services of an expert medical man, are considering the further steps to be taken.

### Cocaine for Use in Dentistry.

The Home Secretary stated that the Committee appointed to consider the question of authorisations for the use of cocaine in dentistry had reported, and the findings would be issued shortly. In view of some questions raised, the general permit to unregistered dentists as to the use of 1 per cent. cocaine would be extended for two months.

### Mentally Deranged Sailors and Soldiers.

Sir William Byles, in a question to the Under-Secretary of State for War, alluded to a protest by the Lancashire Asylums Board against sailors and soldiers, returning home mentally deranged and sent to asylums, having their maintenance charged to boards of guardians as if they were pauper lunatics. Mr. Barnes, the Minister of Pensions, said that it was hoped that shortly an improvement would be effected in the arrangements for dealing with these cases.

### Tuberculosis in Wales.

Sir Edwin Cornwall informed Mr. Haydn Jones that 10,329 persons were examined in 1916 by the tuberculosis physicians in Wales and Monmouthshire, and 4,679 were found to be suffering from tuberculosis; the number suspected and kept under observation was 1,635, and institutional treatment was given to 3,827; none are on the waiting list for sanatorium benefit.

### Pharmaceutical Chemists with Colonial Diplomas.

The Home Secretary informed Sir William Collins that by-laws were made by the Pharmaceutical Society and approved by the Privy Council in 1912, providing for the registration, under certain conditions, of holders of Colonial certificates. No by-laws have yet been made in respect of military dispensers or certified assistants to apothecaries, though the Pharmaceutical Society has been strongly urged by the Privy Council Office to give effect to the intentions of Parliament in the matter.

### Naval Hospital Ships.

Dr. Macnamara informed Commander Bellairs that none of the merchant ships taken up as naval hospital ships are being employed as fixed hospital ships, and that the question of releasing these ships and using shore hospitals had been most carefully considered by the naval authorities, and it had been decided that no change could be made without materially interfering with the Fleet and the proper care of the sick and wounded officers and men.

